# Early movement restriction leads to maladaptive plasticity in the sensorimotor cortex and to movement disorders

**DOI:** 10.1038/s41598-018-34312-y

**Published:** 2018-11-05

**Authors:** Maxime Delcour, Michaël Russier, Francis Castets, Nathalie Turle-Lorenzo, Marie-Hélène Canu, Florence Cayetanot, Mary F Barbe, Jacques-Olivier Coq

**Affiliations:** 1 Neurosciences Intégratives et Adaptatives, UMR 7260, CNRS, Aix-Marseille Université, 13331 Marseille, France; 2 Centre de Recherche en Neurobiologie et Neurophysiologie de Marseille UMR 7286, CNRS, Aix-Marseille Université, 13344 Marseille, France; 3 FR 3512 Fédération 3C, Aix Marseille Université – CNRS, 13331 Marseille, France; 4 Université de Lille, EA 7369 « Activité Physique, Muscle et Santé » - URePSSS - Unité de Recherche Pluridisciplinaire Sport Santé Société, 59000 Lille, France; 5 Institut de Neurosciences de la Timone, UMR 7289, CNRS, Aix-Marseille Université, 13385 Marseille, France; 6 Department of Anatomy and Cell Biology, Lewis Katz School of Medicine, Temple University, Philadelphia, PA 19140 USA; 7 Present Address: Equipe de Recherche en Réadaptation Sensorimotrice, Faculté de Médecine, Département de Physiologie, Université de Montréal, C.P. 6128, Montréal, H3C 3J7 Canada; 8 Present Address: Inserm UMR 1072, Unité de Neurobiologie des Canaux Ioniques et de la Synapse, Faculté de Médecine Secteur Nord, 13344 Marseille Cedex 15, France; 9 Present Address: UMR_S1158 Inserm-Sorbonne Université, Neurophysiologie Respiratoire Expérimentale et Clinique, Faculté de Médecine, 75636 Paris Cedex, France

## Abstract

Motor control and body representations in the central nervous system are built, i.e., patterned, during development by sensorimotor experience and somatosensory feedback/reafference. Yet, early emergence of locomotor disorders remains a matter of debate, especially in the absence of brain damage. For instance, children with developmental coordination disorders (DCD) display deficits in planning, executing and controlling movements, concomitant with deficits in executive functions. Thus, are early sensorimotor atypicalities at the origin of long-lasting abnormal development of brain anatomy and functions? We hypothesize that degraded locomotor outcomes in adulthood originate as a consequence of early atypical sensorimotor experiences that induce developmental disorganization of sensorimotor circuitry. We showed recently that postnatal sensorimotor restriction (SMR), through hind limb immobilization from birth to one month, led to enduring digitigrade locomotion with ankle-knee overextension, degraded musculoskeletal tissues (e.g., gastrocnemius atrophy), and clear signs of spinal hyperreflexia in adult rats, suggestive of spasticity; each individual disorder likely interplaying in self-perpetuating cycles. In the present study, we investigated the impact of postnatal SMR on the anatomical and functional organization of hind limb representations in the sensorimotor cortex and processes representative of maladaptive neuroplasticity. We found that 28 days of daily SMR degraded the topographical organization of somatosensory hind limb maps, reduced both somatosensory and motor map areas devoted to the hind limb representation and altered neuronal response properties in the sensorimotor cortex several weeks after the cessation of SMR. We found no neuroanatomical histopathology in hind limb sensorimotor cortex, yet increased glutamatergic neurotransmission that matched clear signs of spasticity and hyperexcitability in the adult lumbar spinal network. Thus, even in the absence of a brain insult, movement disorders and brain dysfunction can emerge as a consequence of reduced and atypical patterns of motor outputs and somatosensory feedback that induce maladaptive neuroplasticity. Our results may contribute to understanding the inception and mechanisms underlying neurodevelopmental disorders, such as DCD.

## Introduction

It is now understood that development of movement repertoires, motor control and body representations in sensorimotor circuitry are achieved through early spontaneous movements, sensorimotor experiences and reafference in children^1–3^ and rodents^4,5^. From this, it seems likely that early atypical sensorimotor experiences in children should lead to the emergence of atypical movements, motor control problems and disorganization of sensorimotor circuitry that persist into adulthood. The impact of disuse or immobilization (i.e., constraint-induced movement therapy) during child development has mainly been studied in the presence of brain lesions^6^. Only a very few studies have focused on the impact of early disuse occurring in the absence of brain damage. For example, swaddling, the ancient practice of wrapping infants in cloth so that limb movements are restricted, appears to delay the onset of several motor skills^7,8^. Swaddling has become popular again, such as in neonatal intensive care units using elastic cloth, as a means to reduce sudden infant death syndrome or crying, promote sleep, or improve muscle tone^8,9^. Disturbances in the planning, execution and control of body movements in the absence of brain damage are now termed developmental coordination disorders (DCD) and usually coexist with various deficits in executive functions in 5–6% of school-aged children^10^. Patients with DCD show reduced abilities to produce consistent movements^11^, poor motor coordination and kinaesthetic acuity^12,13^, broad impairments in sensorimotor representations and perception^13,14^, each reflected by disrupted central networks^15^ Children with autism spectrum disorder (ASD) also exhibit gross or fine motor abnormalities, motor learning deficiencies and difficulties executing sequences of actions^16^. Most children with DCD or ASD show similar sensorimotor impairments, reduced physical activity and interactions with their environment, and atypical motor development, the latter detected as atypical spontaneous or general movements (GMs)^17,18^.

During typical development, the repertoire of GMs in limbs increases over time in variation, fluency, amplitude and complexity into a continuous stream of small and elegant movements. Atypical GMs correspond to rigid, cramped synchronized and stereotyped movements that exhibit limited fluency, variation and complexity with increasing age^3^. Arising from spontaneous, self-generated and evoked movements during maturation, early somatosensory feedback drives electrical activity patterning from the spinal cord to the cortex. This feedback guides the development and refinement of the anatomical and functional organization of sensorimotor circuitry in rodents^5,19,20^. Atypical, disturbed GMs reflect impaired connectivity and functional disorganization in the brain^2,21,22^. Accordingly, we hypothesized that limited and abnormal patterns of somatosensory inputs during development may lead to abnormal anatomical and functional organization of the sensorimotor circuitry in adulthood as a result of maladaptive cortical plasticity. Such sensorimotor disorganization may in turn alter somatosensory and bodily perceptions, motor outputs, and musculoskeletal structure and physiology.

We recently showed that transient postnatal sensorimotor restriction (SMR), experimentally produced using 28 days of transient (16 hours/day) hind limb immobilization of developing rats, lead to degraded locomotion on a treadmill that persisted for more than 30 days after cessation of the immobilization. This long-lasting degradation was characterized by reduced length, amplitude and velocity, overextended knees and ankles, and digitigrade locomotion that resembled true pes equinus or “toe walking”^23^. SMR rats displayed not only increased variations in the kinematic parameters of treadmill locomotion, mentioned above, but also reduced variations of hind limb joint angles; these variations persisted over time^23^. These results appear to recapitulate the “toe walking” and increased spatiotemporal variations of movements observed in children with ASD^24,25^. SMR also led to musculoskeletal histopathology and increased stretch reflexes, suggestive of muscle hyperreflexia and spasticity. We postulated that reduced and atypical patterns of both motor outputs and somatosensory reafference during development likely contributed to the emergence of movement disorders and musculoskeletal pathologies that persisted into adulthood^23^. To further explore this concept that adult movement disorders may have developmental origins even in the absence of brain damage, we investigated here the impact of postnatal SMR on the neuroanatomical and functional organization of hind limb somatosensory and motor representations in adult rats using microelectrode cortical mapping techniques, and brain histology and immunochemistry. We also assessed the balance between excitation and inhibition in the sensorimotor cortex using *in vivo* microdialysis and western blotting methods. To further understand the processes and interactions underlying any maladaptive neural plasticity, we performed principal components analyses (PCA) to summarize the many variables recorded within the same animals and linear correlations between these variables.

## Results

**Figure 1:**
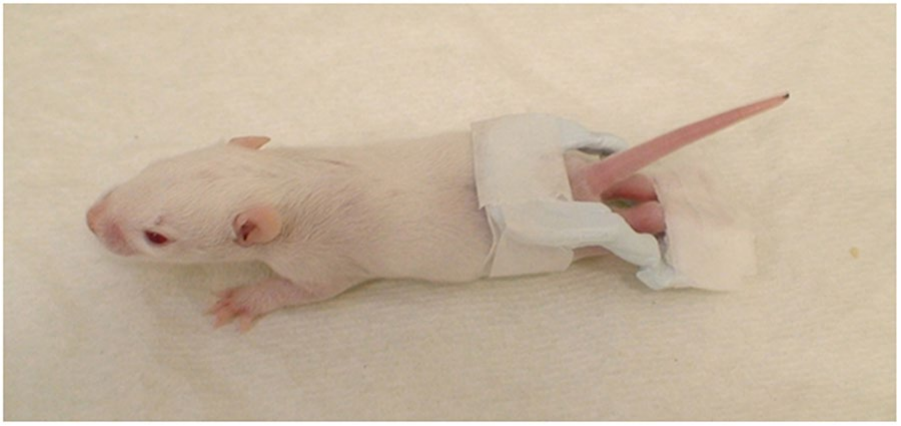
A young rat submitted to postnatal hind limb immobilization leading to sensorimotor restriction (SMR). To restrict movement, the feet of the pup were first tied together with medical tape and then attached to a cast made of epoxy stick. The proximal part of the hind limb was also taped to the cast. The hip joint was free to move perpendicular to the knee, ankle and toe joints that remained in an extended position during the casting period of 16 hours per day from P1 to P28.

We used 17 rats that were exposed to sensorimotor restriction (SMR; Fig. 1) and 20 control rats of either sex. The rats that underwent gait testing at P30 and P65 were also used for hind limb musculoskeletal assessments (results of which have been previously published^23^), as well as electrophysiological procedures (somatosensory maps: SMR, n = 9; Cont, n = 10; neural motor maps: SMR, n = 8; Cont, n = 9), *in vivo* microdialysis (SMR, n = 8; Cont, n = 6) and Western-blotting (SMR, n = 9; Cont, n = 6), and stereological brain histology and immunohistochemistry studies (SMR, n = 13; Cont, n = 18) from P90 to P120, i.e., about 60 to 90 days after cessation of the SMR. Since we did not provide anatomical or histological features of the region of cortex in which we recorded or stimulated, we will refer to somatosensory and motor maps originating from the cortex, as opposed to primary somatosensory and motor cortices^26^.

### SMR degrades the somatosensory map organization and neuronal properties

**Figure 2:**
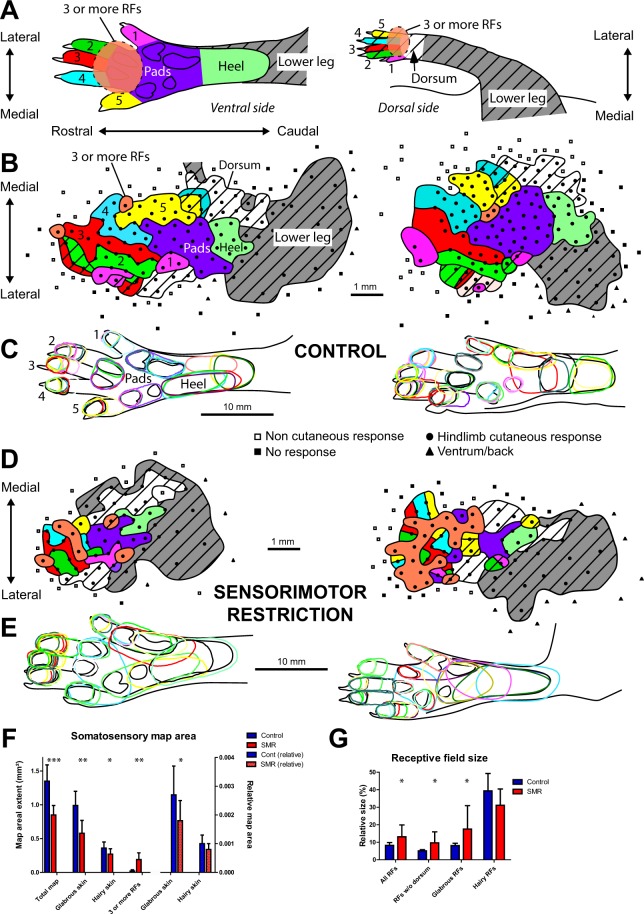
Impact of early sensorimotor restriction (SMR) on the topographical organization of somatosensory map devoted to the skin surface representation of the hind limb in control and SMR rats. (**A**) Illustration of the color code for the somatosensory maps depicted in (**B**), whose cortical sites display receptive fields (RFs) located on the different skin subdivisions of ventral *(left)* and dorsal (*right)* regions of the hind limb, e.g., toes 1 to 5, plantar pads, heel and leg (see Supplementary Methods for details). The hatched zones correspond to cortical sites whose RFs are located on hairy skin surfaces of the hind limb. Sectors in orange correspond to cortical sites whose RFs are located on three or more skin subdivisions of the foot. (**B**) Representative somatosensory maps serving the cortical representation of hind limb skin surfaces in two control rats. All penetration sites of the microelectrode are depicted by squares, circles or triangles. Note the somatotopic organization despite idiosyncratic differences. (**C**) Illustration of all RFs located on glabrous hind paw surfaces corresponding to somatosensory maps from the same control rats shown in (**B**). (**D**) Somatosensory hind limb maps in two representative SMR rats. Note the reduction of the extent of the somatosensory maps after SMR, concomitant to an increase in the size of glabrous RFs illustrated for the same SMR rats shown in (**E**). Changes in map areal extent and RF size are plotted in (**F** and **G**), respectively. (**E**) Illustration of glabrous RFs corresponding to somatosensory maps from the same SMR rats depicted in (**D**). (**F**) Plots of the areal extent of the different subparts of somatosensory hind limb maps in absolute areas (mm²; left Y axis) or normalized values relative to the ventral or dorsal foot skin surfaces (right Y axis). (**G**) Plots of the size of all RFs and their subcategories that correspond to those illustrated in (**F**). Mean + SD. ^*^p < 0.05; ^**^p < 0.01; ^***^p < 0.001, significantly different from controls.

As shown in previous studies^27,28^, the somatosensory hind paw representation originating from the cortex displayed invariant organizational features despite inter-individual differences, called somatotopy. Briefly, the hind paw representation was usually located medial to the forepaw map and rostral to the tail and back/ventral representations. From rostral to caudal, the somatotopic organization corresponded to the progression of cortical sites from the toes, plantar pads of the sole to the heel, and leg (Fig. 2A,B). From lateral to medial in the rostral portion of the hind paw map, the toes were topographically represented from toe 1 to toe 5 (Fig. 2A–C). The hairy representation of the toes was generally located medial (toe 1 to toe 3, innervated by the saphenous nerve) and lateral (toe 3 to toe 5, innervated by the sciatic nerve) to the glabrous representation of the toes (innervated by the sciatic nerve).

Compared to control rats, the overall somatotopy of the foot maps was degraded in adult SMR rats that had experienced daily hind limb immobilization for a month postnatally. Specifically, the somatosensory representation of contiguous skin surfaces of the foot was dramatically disrupted in SMR rats relative to control rats (Fig. 2B,D). The total area of the somatosensory hind paw representation was 1.6 times smaller in SMR rats than in controls (t = 5.35; df = 17; *p* < 0.0001; Fig. 2F). Analyzed together, the cortical neural areas serving the glabrous and hairy foot surfaces were smaller in SMR rats than in controls (Wilks: F(2,33) = 3.81; p < 0.04), as were the normalized neural areas of both glabrous and hairy foot surfaces (Wilks: F(2,33) = 3.76; p < 0.05; Fig. 2F). Interestingly, the normalized glabrous area was reduced by SMR (t = 2.36; df = 17; *p* < 0.03), whereas the normalized hairy representation of the foot did not differ between the two groups (t = 1.28; df = 17; *p*: n.s.; Fig. 2F), suggestive of a specific effect of developmental SMR on the somatosensory representation of glabrous foot skin surfaces.

Illustrative of changes in neuronal properties after postnatal SMR, the proportion of cortical sites with multiple receptive fields (RFs) (i.e., located on three or more disjunctive foot territories) was 10 times greater in SMR rats (30.8%; 99/322) than in control rats (3.1%; 22/702). In contrast, the percent of double RFs did not differ between SMR (24.5%; 79/322) and control (34.5%; 242/702) rats. Concomitantly, the proportion of single RFs located on a single hind paw location was decreased in SMR rats (44.7%; 144/322), compared to controls (62.4%; 138/702; χ² = 194.57; *p* < 0.0001). In addition to the increased number of multiple RFs after SMR, their size also increased (Fig. 2C,E). The mean size of all RFs was greater in SMR rats than in controls (t = −2.22; df = 17; *p* < 0.04; Fig. 2G). When the very large dorsal foot RFs were discarded to reduce variation, the RFs were approximately twice as large in SMR rats as in controls (t = −2.30; df = 17; *p* < 0.03; Fig. 2C,E,G). Also, the size of glabrous and hairy RFs differed significantly between both groups of rats (Wilks: F(2,33) = 4.06; *p* < 0.03). Specifically, RFs located on glabrous foot skin surfaces were about twice as large in SMR rats as in controls (t = −2.22; df = 17; *p* < 0.04), whereas the size of hairy foot RFs did not differ significantly between the two groups (Fig. 2G), confirming a specific impact of SMR on glabrous foot surfaces. These enlargements lead to overlapping glabrous RFs and less specific foot representations in SMR rats (Fig. 2D,E).

Figure 2D illustrates the patchy representation of glabrous and hairy foot surfaces after SMR. There were more RFs encompassing both glabrous and hairy foot skin surfaces in SMR rats (9.3%; 30/322) than in controls (2.4%; 17/702; χ² = 23.97; *p* < 0.0001), more multiple RFs located on three or more foot subdivisions, and numerous RFs located on both sides of the hind paw. In fact, the delineations between toe and plantar pad representations were difficult in SMR rats, so that map sites with three or more RFs had to be pooled together in specific map sectors (colored in orange in Fig. 2B,D) of the somatosensory foot maps. The map sectors with three or more RFs were much larger in SMR than in control rats (Cont: 0.02 ± 0.02 mm²; SMR: 0.19 ± 0.14 mm²; t = −3.89; df = 17; *p* < 0.001) and the normalized area of such cortical sectors relative to the total map area (expressed as percentages) was about 16 times larger after SMR (Cont: 1.40 ± 1.58%; SMR: 22.22 ± 15.42%; t = −4.26; df = 17; *p* < 0.0005). Thus, SMR induced a drastic degradation of both the topographical organization of the somatosensory hind limb maps and cortical neuronal properties. Interestingly, there was an emergence of RFs covering both sides of the leg in SMR rats (11.8%; 13/110) yet no such double-sided RFs in controls (0%; 0/110; χ² = 13.94; p < 0.0002). However, the area of the somatosensory map devoted to the leg representation did not differ significantly between SMR rats (0.49 ± 0.28 mm²) and controls (0.67 ± 0.30 mm²; t = 1.19; df = 13; *p*: n.s. Fig. 2B,D).

Next, neural responsiveness originating from the cortex to light tactile stimulation was qualitatively classified on the basis of the magnitude of the signal-to-noise ratio, as in a previous study^27^ using a three-level scale (weak, clear and excellent responses). The proportion of excellent responses were greater in SMR rats (32.9%; 106/322) than in controls (12.8%; 90/702), the percent of clear responses decreased in SMR rats (47.8%; 154/322) compared to controls (70.1%; 492/702; χ² = 61.24; *p* < 0.0001), while weak responses did not differ between SMR rats (19.3%; 62/322) and controls (17.1%; 120/702). SMR seemed to increase the neural responsiveness to tactile stimulation, indicative of changes in neuronal properties and increased excitability in the somatosensory hind limb representation.

Thus, SMR favored the emergence of numerous multiple, disjunctive and enlarged RFs, and a decrease in the hind limb map area, resulting in degradation of the somatotopy and topographical organization of the somatosensory cortex, as well as altered neuronal properties. SMR induced-map changes appeared to be specific to glabrous foot skin areas, and the appearance of RFs covering both sides of the hind limb or foot is indicative of degraded neuronal properties after SMR.

### SMR-related alterations of hind limb motor maps

**Figure 3:**
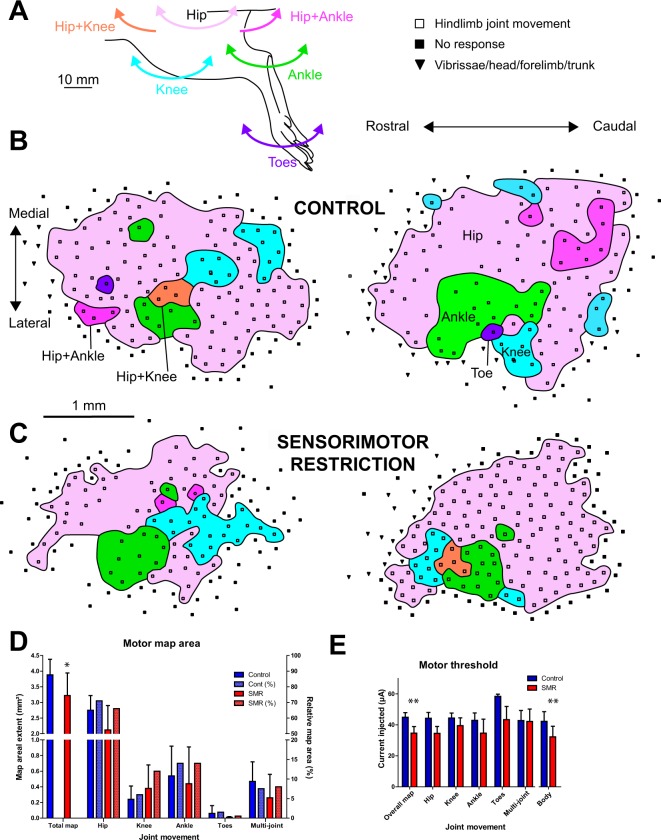
Effects of postnatal sensorimotor restriction (SMR) on the musculotopic organization of the motor representation of hind limb movements in control and SMR rats. (**A**) Illustration of movements of different hind limb joints elicited by intracortical stimulation. The color code for movements of each joint corresponds to motor map sectors devoted to the corresponding movement. (**B**) Typical motor hind limb maps in two representative control rats. Note the patchy representation of hind limb movements and the lack of musculotopy of M1 maps. (**C**) Motor maps of hind limb movements in two representative SMR rats. Note the reduction of the extent of the M1 map after SMR, concomitant to a decrease in the overall threshold needed to evoke movements, changes plotted in (**D** and **E**), respectively. (**D**) Plots of the areal extent of the different subparts of motor hind limb maps in absolute areas (mm²; left Y axis), or relative areas of joint movements normalized to the total area of the hind limb motor map (%, right Y axis). (**E**) Plots of the motor thresholds (µA) that correspond to the minimal current needed to evoke a leg joint movement. Mean + SD. ^*^p < 0.05; ^**^p < 0.01, significantly different from controls.

The representation of hind limb movements around joints did not appear topographically organized but scattered throughout the hind limb motor map (Fig. 3A,B), as previously reported^28,29^. In control rats, the motor hip representation occupied on average 70.9% of the total area of the hind limb motor map (3.88 ± 0.50 mm²; Fig. 3D), while 6.2% was knee, 13.9% was ankle, 1.5% was toes and 7.5% was devoted to complex movements involving several joints of the leg simultaneously. After SMR, we found a reduction in the total area of hind limb motor maps (t = 2.29; df = 15; *p* < 0.04; Fig. 3C,D). Analyzed together, the motor areas devoted to movements of the hip, knee, ankle, toes and multi-joints did not differ between either group (Wilks: F(5,11) = 1.11; *p*: n.s.; Fig. 3C,D). The relative areas devoted to the different joint representations relative to the total motor map area (expressed as percentages) were not significantly altered (χ² = 0.10; p: n.s.; Fig. 3D).

The overall threshold (i.e., minimal amount of current to evoke a movement) of neuronal motor responses was drastically reduced after SMR (Wilcoxon = 2.60; *p* < 0.007; Fig. 3E). Analyzed together, thresholds needed to evoke movements of joints of the leg did not differ between both groups of rats (Wilks: F(5,12) = 1.18; *p*: n.s.; Fig. 3D). Interestingly, the mean threshold required to elicit whisker, neck and forepaw movements outside of the boundaries of the motor hind paw map was significantly reduced (Wilcoxon = 2.19; *p* < 0.03; Fig. 3E), so that SMR appeared to exert a nonspecific role on the motor threshold changes in general. Although the relative representation of each joint within the motor map did not change in SMR rats, the total area for hind limb motor maps was drastically reduced. In addition, the reduction of the minimal stimulation thresholds after SMR revealed an increase in motor excitability.

### SMR changes the balance between glutamate and GABA in the sensorimotor cortex

**Figure 4:**
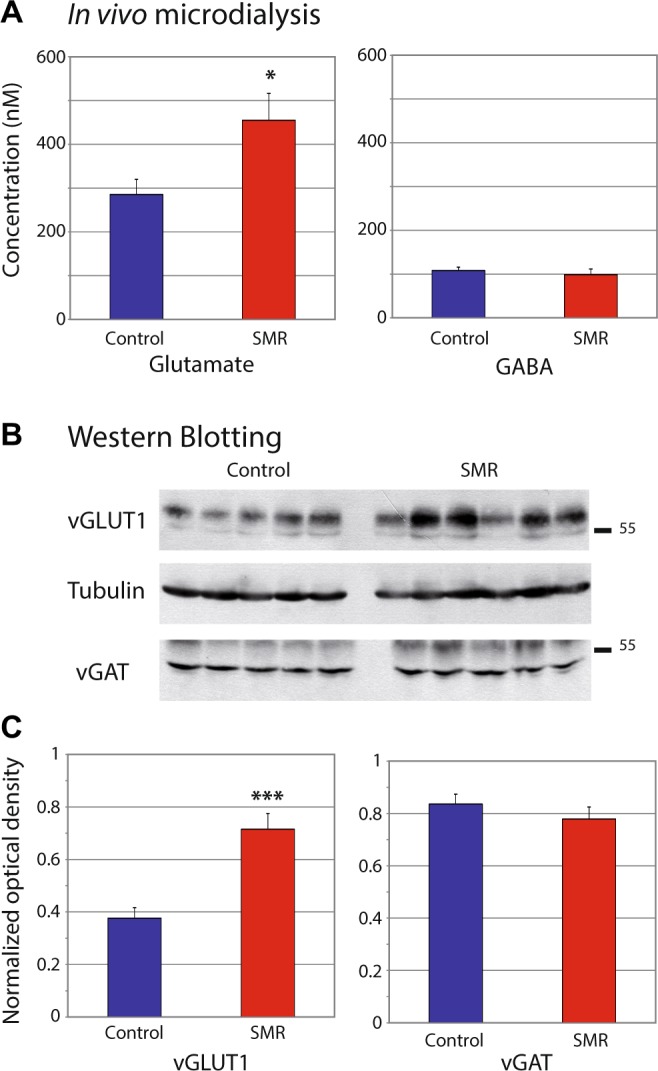
Effects of postnatal sensorimotor restriction (SMR) on the excitation/inhibition balance in the hind limb area of the sensorimotor cortex. (**A**) Plots of glutamate and GABA extracellular levels assessed using *in vivo* microdialysis. (**B**) Typical immunoblots for vGLUT1 (vesicular transporter of glutamate) and vGAT (vesicular transporter of GABA) in the two groups of rats. Tubulin controls show that loading was equal for each lane. (**C**) Plots of glutamate and GABA intracellular levels assessed by Western blots of vGLUT1 and vGAT, respectively. Note that both measures indicate an increase in glutamate levels while GABA remained unchanged after SMR, compared to controls. Mean ± SEM. ^*^p < 0.05; ^***^p < 0.001.

To assess the balance between excitation and inhibition in the hind paw sensorimotor cortical region, we analyzed dialysates obtained during *in vivo* microdialysis for levels of extracellular glutamate and GABA using gradient HPLC coupled to laser detection. The extracellular concentration of glutamate was greater in SMR rats than in controls (t = 2.20; df = 12; *p* < 0.05); whereas, the extracellular concentration of GABA did not differ between the two groups (Fig. 4A).

We then performed semi-quantitative Western blots from brains of additional animals, using specific antibodies against vesicular glutamate transporter (vGLUT1) and vesicular GABA transporter (vGAT), reliable markers of excitatory and inhibitory transmission, respectively. The amount of vGLUT1 increased approximately 90% in SMR rats relative to controls (Wilcoxon = 44; *p* < 0.0004; Fig. 4B,C). In contrast, the amount of vGAT did not differ between the two groups (Wilcoxon = 29.5; *p* = 0.34, n.s.; Fig. 4B,C). Thus, SMR seemed to induce an increase in glutamate release in the sensorimotor cortex of adult rats while GABAergic levels appear unaltered.

### SMR did not induce brain histopathology

No microscope evidence of histopathology was observed in the somatosensory, motor and cingular cortices or underlying white matter, including in the corpus callosum of any SMR rat compared to control rats. Also, there were no differences between the two groups with respect to APP, caspase 3 or GFAP immunoreactivity, as shown in Supplementary Fig. 1. Nor were there differences in Fluoro-Jade staining that would be indicative of axonal injury in the brains or spinal cords of either group (data not shown).

The numerical density of NeuN positive cells did not differ significantly between the two groups of rats in either the hind paw somatosensory representation (Cont: 99.3 ± 22.4 × 1000/mm^3^; SMR: 86.8 ± 9.5 × 1000/mm^3^; Wilcoxon = 12; p = 0.71, n.s.) or hind paw motor representation (Cont: 96.7 ± 19.3 × 1000/mm^3^; SMR: 92.0 ± 16.9 × 1000/mm^3^; Wilcoxon = 12; p = 0.71, n.s.). In the same line, the numerical density of GAD positive cells did differ not between the two groups of rats in either the hind limb somatosensory representation (Cont: 7.0 ± 2.4 × 1000/mm^3^; SMR: 7.8 ± 2.4 × 1000/mm^3^; Wilcoxon = 7.5; p = 0.62, n.s.) or the hind limb motor representation (Cont: 7.0 ± 1.8 × 1000/mm^3^; SMR: 6.3 ± 2.2 × 1000/mm^3^; Wilcoxon = 13; p = 0.54, n.s.). Thus, SMR appeared to have no significant impact on brain neuroanatomy, compared to controls.

### Functional relationships between sets of variables using PCA and correlations

In the present study, 105 variables obtained from the same rats (Cont: n = 18; SMR: n = 16) were used to describe 7 sets of variables: somatosensory (n = 23 variables) and motor (n = 17) hind limb map features, neurotransmission (n = 4) and neurohistopathology (n = 6), as well as locomotion at P30 (n = 24) and P65 (n = 24) and musculoskeletal histopathology (n = 7) as previously reported^23^. In that study, locomotion on a treadmill was assessed at P30 and P65, as was musculoskeletal histopathology in these same rats (see data from all variables in Supplementary Table 1).

First, to define the variables that contributed the most to differentiating the two groups of rats, we computed a PCA to compress the dataset into a single score for each rat, based on coordinates along a PC (Supplementary Table 1). The overall score along PC1 summarized 24.4% of the variance of the dataset. The mean score for SMR rats (−4.1 ± 4.0) differed significantly from the score for controls (3.7 ± 1.8; Wilcoxon = 323; *p* < 0.0001), indicating a clear separation of the two groups along PC1 (Supplementary Fig. 2A). The highest contributing variables included: 1) locomotion kinematics and joint angles at P30 (15 variables out of 24, including 9 variables with factor loadings along PC1 ≥ |0.76|), 2) locomotion at P65 (16/24 variables including 3 variables with factor loadings ≥ |0.76|), 3) somatosensory hind limb map features (14/23 variables including 7 variables |0.53| < factor loadings < |0.73|), and 4) musculoskeletal histopathological changes (6/7 variables including 2 variables with factor loadings ≥ |0.62|). In contrast, variables for motor hind limb map features (2/17 variables including the smallest factor loadings = |0.45|), neurotransmission (1/4 variables) and neurohistopathology (0/6 variables) appeared to contribute little to changes observed in PC1 (see Supplementary Table 1).

Second, to evaluate the relationships between the 7 sets of variables, individual animal scores for each set of variables (Supplementary Fig. 2B) were compared. We observed that somatosensory hind limb map features were highly correlated to locomotion kinematics at P30 (r = 0.84; *p* < 0.0002) and P65 (r = 0.84; *p* < 0.0007), to musculoskeletal histopathology (r = 0.75; *p* < 0.0001) and tended to correlate with neurotransmission findings (r = 0.54; *p* = 0.08; Table 1). Surprisingly, the neurotransmission variables correlated with musculoskeletal histopathology (r = 0.51; *p* < 0.04) and somatosensory map features, but not with the other sets of variables. The motor hind limb map features along the PC2 axis significantly correlated to the highest contributing variable, which was locomotion at P30 (r = 0.53; *p* < 0.03), and to musculoskeletal histopathology (r = 0.50; *p* < 0.04), but not to the other sets of variables (Table 1). Musculoskeletal histopathology was highly correlated with locomotion at both P30 (r = 0.75; *p* < 0.0001), as well as at P65, although to a lesser level (r = 0.56; *p* = 0.003). In contrast, locomotion features at both ages were highly interrelated (r = 0.82; *p* < 0.0001), suggestive of a self-perpetuating interplay between these variables, as described previously^23^. Neurohistopathological variables did not correlate significantly to any other variables, confirming the lack of impact of SMR on brain neurohistology. Unfortunately, we could not assess the relationships between somatosensory and motor map features as we could not map both cortices in the same animals due to the use of different anesthetics.

**Table 1:** Correlations between the 7 sets of variables, based on the individual scores along PC1 (x-axis) or PC2 (y-axis) of animals obtained by using PCA. Pairwise two-sided p-values: ^a^*p* < 0.05; ^b^*p* < 0.001; ^†^tendency, p = 0.08.

	Somatosensory map features PC1	Motor map features PC2	Neurotransmission PC1
Locomotion P30 PC1	**0**.**84**^**b**^	**0**.**53**^**a**^	0.30
Locomotion P65 PC1	**0**.**84**^**b**^	0.06	0.27
Musculoskeletal pathologies PC1	**0**.**75**^**b**^	**0**.**50**^**a**^	**0**.**51**^**a**^
**Neurotransmission PC1**	***0***.***54***^†^	0.28	
**Neurohistopatho.** **PC1**	0.36	0.43	0.19

Thus, the most contributing variables differentiating SMR rats from controls were locomotion at P30 and P65, then somatosensory map features, as well as the circumference and belly length of the gastrocnemius (indicative of muscle atrophy). SMR-related somatosensory map reorganization was highly related to locomotion impairments, excitation/inhibition imbalance and musculoskeletal histopathology. Surprisingly, motor map features and neurotransmission contributed the least to group differentiation. Reorganization of the motor hind limb representation was also related to locomotion impairments, although only at P30, and to histopathology, although to a lesser extent than the degradation of the somatosensory hind limb representation. Peripheral histopathology and locomotion impairments were highly correlated, confirming the self-perpetuating interplay between these variables.

We also found that sex had no specific impact on the features explored here and previously^23^.

## Discussion

To investigate a hypothesis of a developmental origin of adult disorders, we showed in the present study that reduced and abnormal patterns of somatosensory inputs after hind limb movement restriction during postnatal development had no significant impact on adult brain neuroanatomy yet did effect the functional organization of the sensorimotor cortex. Compared to controls, SMR led to: 1) a degradation of both somatosensory hind limb map organization and neuronal properties; 2) a decrease in the total motor map area devoted to hind limb movements concomitant with an overall increased excitability; 3) increased glutamate release, yet no changes in GABA levels in the sensorimotor cortex; yet 4) no neuroanatomical changes in the sensorimotor hind limb cortex.

To evaluate interactions underlying the observed neural maladaptive plasticity, we used PCA and correlational statistics. We found that SMR primarily effected locomotion at P30 and P65, as well as somatosensory map features and musculoskeletal features in decreasing order; yet had lesser effect on motor map features, neurotransmission or neurohistology. This meta-analysis of all data from the same rats also outlines that: 1) deterioration of somatosensory maps is tightly related to locomotion impairments, neurotransmission imbalance and musculoskeletal histopathology; 2) locomotor disorders and musculoskeletal pathology likely interplay tightly in a self-perpetuating cycle (see^23^); whereas, 3) motor map alteration was weakly influenced by these variables and not by neurotransmission.

### Putative mechanisms of plasticity underlying the functional disorganization of the hind limb representations

SMR induced a specific and drastic reduction of the somatosensory representation of the glabrous foot surfaces concomitant with a specific and marked enlargement of glabrous RFs, suggestive of a use-dependent (Hebbian) process of plasticity^30,31^. The emergence of cortical sites with three or more disjunctive RFs and/or RFs located on both glabrous and hairy sides of the foot or leg in adult rats led to a greater overlap between glabrous RFs, a coarser-grained representation of the somatosensory foot representation. Most importantly, this emergence led to a dedifferentiation of the cortical representation of both single skin territories, such as toes and plantar pads, and glabrous/hairy surfaces that are usually represented segregated in control rats^27^. In sum, SMR degraded the overall somatotopy and fine-detailed topography of the somatosensory hind limb representation and neuronal properties that correspond to degraded abilities in tactile discrimination^32^. SMR induced a decrease in the motor leg map area while the relative cortical areas serving the different joint representations were unchanged, so that the overall “musculotopy” of hind limb movements was maintained despite inter-individual variations. Another key result after SMR was the decrease in the overall threshold of neurons in the motor cortex needed to evoke leg movements, a decrease that was not specific to the leg area. This threshold decrease corresponded to an overall increase in motor cortex excitability that fits with both increased cortical neuron responsiveness to tactile stimulation after SMR and increased glutamatergic transmission, yet unchanged GABA levels in the sensorimotor leg area. These results confirm and extend prior studies using similar hind limb immobilization during development^27,29,33^, in which reorganization originating the sensorimotor cortex was extensively discussed, although the literature about chronic activity deprivation during development is limited.

We investigated the excitation-inhibition neurotransmission in the sensorimotor cortex because it appears to govern cortical map reorganization. Use-dependent regulation of glutamatergic excitation underlies expansion or retraction of somatosensory maps, while use-dependent GABAergic inhibition promotes cutaneous RF changes, corresponding to the modulation of neuronal tuning on the basis of synaptic potentiation/depression and unmasking/masking of thalamocortical divergent and convergent connections, respectively^30,31,34^. In the same line, activity-dependent changes in excitation-inhibition balance leads to masking or unmasking of intracortical motor connections that acts upon motor map boundaries and thresholds, i.e., neuronal tuning^35,36^. Decreased hind limb sensorimotor maps and increased size of RFs did not match with the overall hyperexcitability found in the hind limb sensorimotor area, so that the hind limb map reorganization may be explained by alternative mechanisms of plasticity. The absence of neuroanatomical pathology after SMR allows us to rule out its impact on the neurotransmission changes and map reorganization. In addition, SMR induced a long-lasting increase in the stretch reflex evaluated with post-activation depression^23^, that is supposed to reflect hyperexcitability within the lumbar spinal cord and spasticity^37,38^. The latter finding fits with the present observation of enduring cortical hyperexcitability.

In this long-term study, a slow homeostatic (non-Hebbian) increase in global synaptic strength (i.e., synaptic scaling) and/or intrinsic excitability, which in turn enhances responses to remaining inputs^31,39^, may explain the increased cortical excitation observed in adult SMR rats. An alternative explanation for such an increase could reside in the persistence of dense dendritic arborisation that are usually pruned during the first few postnatal weeks^40^. For instance, postnatal whisker removal reduces the pruning of supernumerary dendrites that are normally present in S1 layer V pyramidal cells^41^, thus preserving abnormally important levels of excitatory synapses involved in increased cortical excitability^42^. Thus, a co-existence of both Hebbian and homeostatic processes of plasticity may explain the observed differences in remodelling of somatosensory and motor maps in relation to hyperexcitability in the sensorimotor cortex.

### Putative interactions and processes underlying maladaptive plasticity

Based on PCA and correlations within the same animals, our meta-analysis indicates that the SMR-related deterioration of the somatosensory map organization and neuronal properties was tightly related to locomotor disorders evaluated at P30 and P65. SMR led to severely degraded kinematics of gait on a treadmill in relation to knee and ankle overextension, and smaller footprints that revealed a digitigrade locomotion^23^, which resembles “toe walking” or true pes equinus, observed in children with ASD^24^. According to the literature^30,31,43,44^, typical plantigrade locomotion in control rats^23,45^ produces asynchronous and distributed stimulation through the foot skin surfaces from proximal to distal parts during the stance up to the beginning of the swing. During typical motor development, spatiotemporally distributed cutaneous inputs of the foot in controls likely contribute to the differentiated representation of the different foot skin surfaces, such as individual toes and plantar pads. In contrast, we postulate that digitigrade locomotion during postnatal SMR provides synchronous and colocalized tactile inputs from several toe and/or pad surfaces, leading to enlarged RFs that cover abnormally large skin surfaces. This enlargement results in the dedifferentiation of the somatosensory representation of contiguous foot territories and finally to the disruption of the somatotopic and topographic organization of the somatosensory foot maps, also observed in animal models of focal hand dystonia^44,46^. In addition, unrepresented skin surfaces in control rats, such as the proximal phalanges of toes and skin surrounding pads, became represented in the somatosensory maps after SMR. The very large RFs covering the entire leg of SMR rats may result from synchronous activations of cutaneous mechanoreceptors during chronic co-contractions of antagonist leg muscles (see^27^). Taken together, abnormal, specific tactile stimulation during digitigrade locomotion induced a maladaptive reorganization in the somatosensory cortex, in which only the glabrous representation was altered by SMR, not the hairy representation (see^27^). Neural maladaptive plasticity of the somatosensory maps also appeared highly related to musculoskeletal histopathology and neurotransmission.

**Figure 5:**
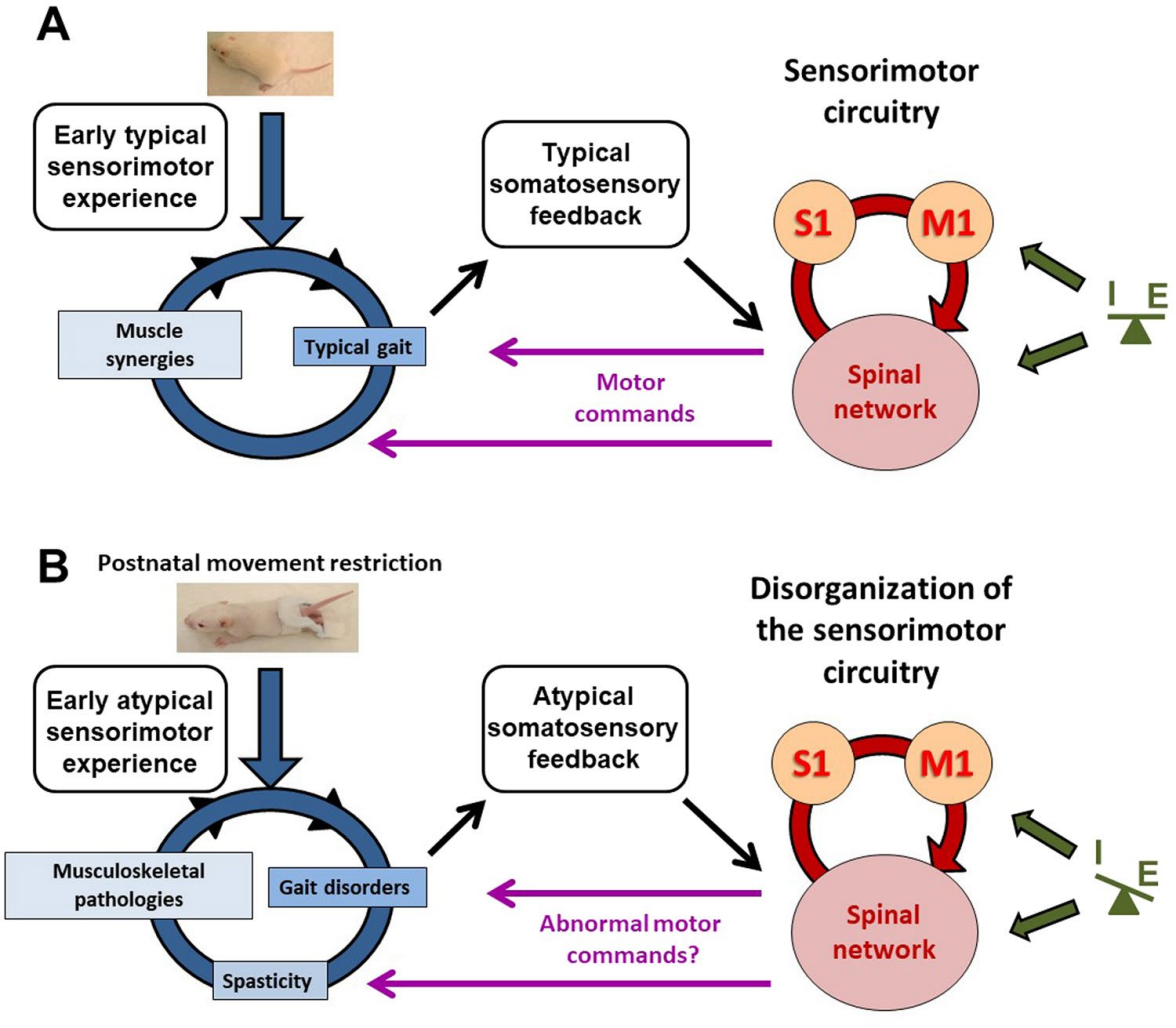
Illustration of the interactions and processes underlying typical or atypical sensorimotor development in rats. (**A**) Schematic representation of the putative interactions between the different variables assessed (locomotion, musculoskeletal tissues, Hoffman-reflex, somatosensory and motor representations of the hind limb and excitation-inhibition neurotransmission in the sensorimotor cortex) that may occur during typical sensorimotor development in control rats. Daily activities provide early typical sensorimotor experience that allow muscle synergies and typical gait to interplay harmoniously. Normal movements during typical gait produce typical sensorimotor feedback to the immature brain, contributing to the development and refinement of connections, maps and functions, e.g., normal motor command to muscles that in turn contribute to muscle synergies and typical movements in a virtuous self-perpetuating cycle. Excitation-inhibition balance also participates in the refinement of neural maps and connections. (**B**) Schematic illustration of the deleterious impact of postnatal sensorimotor restriction (SMR) on neuromuscular interplay. Movement restriction during postnatal hind limb immobilization provides early atypical sensorimotor experience that leads to gait disorders, musculoskeletal pathologies and hyperreflexia, as signs of spasticity (see^23^). In turn, gait disorders and spastic muscles provide atypical somatosensory feedback/reafference to the immature sensorimotor circuitry, mainly the spinal network and the primary somatosensory (S1) and motor (M1) cortices that intercommunicate. Such atypical somatosensory feedback likely degrades the functional organization of the somatosensory and motor maps and spinal network, thus producing abnormal motor commands that finally aggravate gait disorders, spasticity and musculoskeletal abnormalities into interrelated self-perpetuating cycles. Excitation-inhibition imbalances that tip the balance to hyperexcitability in sensorimotor hind limb cortices have more impact on the reorganization of somatosensory maps than on motor maps. Hyperexcitability in the lumbar spinal cord has been shown previously as an enduring hyperreflexia in adult SMR rats (see^23^).

Those correlations do not indicate a directionality of the relation, so that it means that locomotor disorders and musculoskeletal histopathology may be attributed to the somatosensory map degradation. Yet, the high correlation between locomotion degradation and musculoskeletal histopathology after SMR confirms their interplay in a self-perpetuating cycle (Fig. 5), in which the spasticity related to increased stretch reflex also contributed to this cycle^23^. Surprisingly, only locomotion disorders at P30 and musculoskeletal histopathology correlated weakly with the motor map reorganization. In addition, neurotransmission appeared correlated primarily with somatosensory map features and secondarily with musculoskeletal pathologies, but not with motor map features or locomotion. Taken together, our results emphasize the key role of the functional degradation of the somatosensory hind limb representation, which appears to mainly depend on abnormal patterns (quality) of somatosensory inputs resulting from both postnatal SMR and enduring degraded reafference resulting from disturbed locomotion rather than decreased levels (quantity) of inputs, which likely seems involved in the excitation/inhibition imbalance^47,48^. These results proceed from dynamic selection, competition and collaboration of primary neuronal groups on the basis of afferent inputs resulting from behaviors and experience that induce changes in the strength of synaptic connections, thus governing the topographic organization of cortical maps and the corresponding tuning of neurons, and leading to diverse repertoires of movements^49–51^. Thus, the development and continuous refinement of neural connectivity is influenced interactively by feedback from neural targets^52^, supporting the interplay between neural connections, maps and functions intermingled in regulatory loops and self-perpetuating cycles sustained by experience.

Therefore, we postulate that early limited and abnormal patterns of movements through postnatal hind limb immobilization during the light active period provide atypical sensorimotor experience at the origin of adult locomotor disorders (e.g. digitigrade gait), increased stretch reflex (i.e., lumbar spinal cord hyperexcitability, spasticity, spasms, contractures…) and musculoskeletal tissue degradation, compared to typical sensorimotor development in controls (Fig. 5^23^). Our meta-analysis suggests the existence of a self-perpetuating cycle, in which musculoskeletal pathologies likely contribute to muscle overactivity and locomotor disorders. Spasticity and related muscle disorders may contribute in turn to enhanced musculoskeletal and locomotion disturbances (Fig. 5^23^). As a consequence, degraded movements due to spastic muscles and digitigrade locomotion likely produce atypical somatosensory feedback to the immature sensorimotor circuitry, including the lumbar spinal circuitry involved in automatic gait, and primary somatosensory and motor cortices associated with other subcortical structures in the sensorimotor loop^19^. In the absence of brain damage, this atypical somatosensory reafference probably triggers and maintains the functional disorganization of the sensorimotor maps and neuronal properties, and likely that of the lumbar spinal cord (i.e., hyperexcitability related to increased stretch reflex), involved in both motor commands during automatic locomotion and the emergence of muscle overactivity and paresis (Fig. 5^53,54^). The disorganized sensorimotor circuitry may in turn produce atypical or aberrant motor commands, such as abnormal muscle synergies involved in gait disorders observed in rats after hind limb unloading^55^ or in patients with stroke^56,57^. Thus, we propose the existence of three interplaying, self-perpetuating cycles: the first cycle is related to peripheral changes, including gait disorders, increased stretch reflex and musculoskeletal pathologies (Fig. 5). The second cycle involves the sensorimotor circuitry. The third one corresponds to interactions between the peripheral and central circuitry that are interconnected by the somatosensory reafference (altered by abnormal movements) and atypical motor commands that drive abnormal movements (Fig. 5).

### Functional implications

The present study based on postnatal SMR confirms and emphasizes the preponderant role of individual, early experience in shaping the body and brain, and modulating their interplay from maturation to adulthood, depending on physical and environmental constraints^4,19,52^. Limited and abnormal patterns of somatosensory inputs arising from SMR in early development, in the absence of neuroanatomical histopathology, induced long-lasting locomotor disorders, hind limb musculoskeletal histopathology, hyperreflexia and functional disorganization of the adult sensorimotor cortex. Taken together, this recapitulates several symptoms and neurological features found in patients with ASD, and DCD in general^10,18,22^. Adults with DCD that have no evident brain damage, show reduced abilities to produce consistent movements^11^ and impairments in kinesthesic acuity^13^. Children with ASD also exhibit gross and fine motor abnormalities, motor learning deficiencies, inabilities in executing sequences of actions^16^ and deficits in tactile localization that may reflect broad impairments in multisensory body representations^13–15^. Thus, these children share early aberrant sensorimotor experience resulting from atypical spontaneous or general movements (GMs) observed in children with DCD^2,17,58^, along with its related brain disorganization, especially inabilities in processing and integrating sensory information^2,13,16^. GMs are predictive of neural, motor and cognitive outcomes in children with DCD^2,10,21,22,58^. Our animal studies on postnatal SMR supports the idea that developmental maladaptive plasticity may lead to long-lasting motor disturbances, as observed in DCD and point out the pertinence of early rehabilitative programs. Further studies are needed for a better understanding of the possible impact of early SMR on behavioral and cognitive abilities and on the postnatal development of the lumbar spinal circuitry.

## Materials and Methods

All experiments and animal use were carried out in accordance with the guidelines laid down by NIH (NIH Publication #80-23) and EC Council Directive (2007/526/EEC). This research involving animals was approved by the Direction Départementale des Services Vétérinaires – Préfecture des Bouches du Rhône, France (permit # C13-055-18).

### Animals and hind limb movement restriction

Sprague-Dawley rat pups of either sex from different litters were pseudo-randomly assigned to two groups: 1) a group subjected to transient hind limb immobilization from P1 to P28 for 16 hours per day, thus producing sensorimotor restriction (SMR); and 2) a control group (Cont). Feet of SMR pups were gently bound together with medical tape. Their hind limbs were then immobilized in an extended position and taped to a cast made of epoxy putty sticks (Fig. 1). The casts were well tolerated by the pups and mothers, and allowed the pups to move at the hip, urinate, defecate and receive maternal care. After casting, pups were returned to their mother and unrestrained littermates during the dark phase, a period of peak motor activity. During the day’s light phase, the casts were removed so that pups could move freely for 8 h/day. Daily, while uncasted, the hind limb joints were passively moved by the investigators through their full range of motion. The size of the casts was adapted to the growth of the rats from P1 to P28 (see^23^ for details).

### Electrophysiological mapping in cortical sensorimotor areas

The topographical organization and neuronal properties of the primary somatosensory (S1) and motor (M1) cortices were examined in rats from 90 to 120 days-old, using either microelectrode electrical recordings or stimulation, respectively. Hind limb maps were derived from the left hemisphere, as these maps correspond to the right contralateral hind limb whose locomotion kinematics were previously recorded and reported^23^.

#### Somatosensory hind limb maps

The somatosensory mapping procedure used was as previously described^27,46^ (for more details, see Supplementary Methods). Briefly, each animal was anaesthetized with sodium pentobarbital (50 mg.Kg^−1^, i.p.); supplemental doses (5 mg.Kg^−1^, i.p.) were provided as needed to maintain a deep and stable level of anaesthesia throughout the mapping session. A craniotomy was made over the S1 hind limb representation located between −1 and + 3 mm from bregma in the rostrocaudal axis and between 1 and 5 mm in the mediolateral direction. Multiunit recordings were made using tungsten microelectrodes (1 MΩ at 1 KHz, WPI, Sarasota, FL, USA) in the upper layer IV (650–700 µm) of the S1 cortex. The low-threshold receptive field (RF) corresponded to the cutaneous area whose barely visible indentation or gentle hair movements elicited clear bursts of S1 neuronal activity. High-threshold responses elicited by taps on the skin were classified as cutaneous responses; stroking of hairs and manipulations of muscles and joints were classified as non-cutaneous responses. Unresponsive cortical sites exhibited spontaneous discharges only. To elaborate maps of the hind limb representation we drew boundaries encompassing cortical sites whose RFs were restricted to a common hind paw subdivision (e.g. toe, plantar pad). When RFs were located on distinct and separate skin subdivisions of the foot, borders were drawn midway between adjacent recording sites. When a single or multiple RF included different but adjoining skin subdivisions of the hind limb, a boundary line was drawn that crossed the cortical sites. Map borders were placed midway between responsive and unresponsive sites (Fig. 2B,D). When RFs were located on three or more disjunctive RFs, we pooled together cortical sites in specific map sectors colored in orange (Fig. 2B,D).

#### Motor hind limb maps

Standard intracortical microstimulation techniques were used to derive detailed maps of hind limb movements^28,29^ (see Supplementary Methods). Animals were anaesthetized with ketamine hydrochloride (70 mg.Kg^−1^, i.p.) and xylazine (5 mg.Kg^−1^, i.p.). Supplemental doses of ketamine (20 mg.Kg^−1^, i.p.) and acepromazine (0.02 mg.Kg^−1^, i.p.) were delivered as needed to maintain a deep and constant level of anaesthesia. A craniotomy was made over the M1 hind limb representation located between −1 and +3 mm from bregma in the rostrocaudal axis and between 1 and 5 mm in the mediolateral direction, indicative of large overlap of somatosensory and motor hind limb maps (See Supplementary Fig. 3). Microelectrodes (100 KΩ, FHC, Bowdoin, ME, USA) were advanced into layer V at a depth of about 1700 µm. At each electrode penetration, stimulation was initiated at the lowest intensity (5 µA) and was increased gradually until lower limb movement was evoked. Movements of the toes, ankle, knee, hip and other joints were detected visually. If no movement was elicited at 60 µA, the site was defined as unresponsive. Maps were reconstructed offline. Detailed mapping procedures are provided in Supplementary Methods.

Somatosensory map areas were described by their absolute areas (mm²) for each rat and normalized relative to either the ventral or dorsal foot skin surfaces for somatosensory maps only. Relative areas of joint movements are normalized relative to the total motor hind limb map area for each rat. Average values were computed for both groups of rats. The absolute sizes of glabrous and hairy receptive fields (RFs), measured in mm², were normalized relative to the ventral and dorsal hind paw skin surfaces, respectively, and expressed as percentages. The relative RF areas measured in each rat were averaged, and mean RF sizes in each group were computed. Mean RF size per rat was computed after discarding the large RFs located on the dorsal surface of the hind paw^27^.

### Excitatory and inhibitory neurotransmission

To gain insight into the long-term impact of SMR on extra- and intracellular levels of glutamate and GABA, in subsets of rats, we performed *in vivo* microdialysis within the sensorimotor cortex located contralateral to the mapped side, at 2 hours before mapping. In other rats, Western blot analysis was used to quantify intracellular amounts of transporters for both glutamate (vGLUT1) and GABA (vGAT) in sensorimotor cortical tissues collected contralateral to the mapped side, at one hour after mapping.

#### *In vivo* microdialysis

Carnegie Medecin microdialysis probes (CMA/11, Phymep, France) were implanted within the hindpaw representation of the right S1-M1 area using stereotaxic coordinates (A: −1/0, L: + 2; H: 2). An implantation site that avoided large blood vessels; traces of blood were not found after probe withdrawal. The terminal ends of the probes were covered with a polycarbonate membrane. The membrane had a diameter of 0.24 mm, a length of 2 mm and a 6 KDa molecular mass cut-off. The membrane acted like a blood vessel, with extracellular molecules passing through it by diffusion gradient. A new probe was used for each rat. This procedure is detailed in Supplementary Methods.

#### Western blotting and quantification

The leg region from the right side of the sensorimotor cortex was collected and homogenized in TBS (Tris-HCl 50 mmol.L^−1^ pH 7.4, NaCl 150 mmol.L^−1^) containing protease inhibitors (complete EDTA free, Roche, Basel, Switzerland) using a Potter homogenizer and a ratio of 100 µg of brain pieces per mL of buffer. Homogenates were centrifuged at 500 g for 3 min at 4 °C. Supernatants were aliquoted and stored at −80 °C until use. Proteins present in each supernatant were resolved by SDS-PAGE and transferred onto nitrocellulose membranes. Proteins of interest were revealed with specific antibodies and then enhanced using chemiluminescence (ECL; Pierce Biotechnology, Rockford, IL, USA). For illustration purposes, image editing was performed using ImageJ software (http://rsb.info.nih.gov/ij/) and was limited to linear brightness/contrast adjustment (see original scans of nitrocellulose membranes in Supplementary Fig. 4). Quantification was performed using ImageJ. Integrative intensities minus background were plotted for each sample after normalization to 1 for the highest value per western blot (see Supplementary Methods).

### Brain immunohistology

After mapping one side of the brain, and microdialysis or collection of tissues for western blot analysis of the contralateral side, the animals received terminal doses of anaesthesia (thiopentobarbital, 150 mg.Kg^−1^), before undergoing transcardial perfusion with 4% paraformaldehyde in phosphate buffer (0.10 M, pH 7.4). The brains were then harvested, postfixed in the same fixative solution for 3 h, cryoprotected in sucrose 30%, and frozen for cryosectioning. Sections to be immunostained for glial fibrillary acidic protein (GFAP), amyloid-ß precursor protein (APP), DAPI (a general cell nucleus marker), NeuN (a neuronal nucleus marker) and GAD (GABAergic interneurons expressing GAD67) consisted of 15 µm coronal sections (see^28^; Supplementary Methods).

Counting of NeuN and GAD-67 cells was performed on the same side of the brain as used for electrophysiology, using a DMR microscope (Zeiss, Oberkochen, Germany) equipped with a motorized stage and Stereo Investigator software-controlled computer system (Microbright-Field Europe, Magdeburg, Germany). Cell counting was performed at x40 magnification using an optical fractionator method in semi serial sections (see Supplementary Methods). In both groups, the number of samples from the left and right hemispheres was roughly equal.

The heights of the genu of the corpus callosum, and S1, M1 and entorhinal cortices were measured in haematoxylin and eosin (H&E) stained sections using a 20x objective and the Auto-Width tool of Bioquant Image Analysis program in which the inner and outer boundaries of structures were traced, and then mean layer thicknesses of a length of genu or cortex were automatically generated. Alternate sections were stained with Fluoro-Jade using previously described methods^28^.

### Data analysis

Data normality and homogeneity of variance were determined with Shapiro test, Bartlett test and var.test before to apply parametric (Welch two sample t test according to interval confidence for independent variables and Wilks one-way MANOVA for dependent variables) or nonparametric (Wilcoxon rank test, Wilcoxon; and chi-square, χ²) tests with normal approximation with continuity correction or correlations (Pearson’s product-moment with normal approximation with continuity correction - Yates correction to the chi-square test – and confidence interval or nonparametric Spearman rank-order with two-sided alternative hypothesis) using only R (The R Foundation for Statistical Computing, Institute for Statistics and Mathematics, Wien, Austria). The investigators were blinded to rearing conditions throughout the different experimental sessions until statistical comparisons were performed.

#### Principal components analysis

The high dimensionality of our dataset (105 variables) made it difficult to easily extract parameters that accounted for SMR-related changes. To identify variables that contributed the most to the overall variability of our data, we used principal components analysis (PCA) to reduce high dimensionality (FactomineR and Psych packages, The R Foundation for Statistical Computing). PCA is defined as an orthogonal linear transformation of a dataset into a reduced subspace with new coordinates, so that the variance is maximized on each new coordinate axis or principal component (PC), thus minimizing the loss of information. A normalization method, in which the data mean was adjusted to 0 and the standard deviation (SD) was adjusted to 1, was applied to allow comparisons of data with disparate values and variances^59^. Then, in order to identify variables that most optimally separate the two groups, we performed a PCA with all dataset variables (n = 105). We usually kept the first two PCs with Kaiser’s criterion over 1 that in combination explained at least 35 to 70% of the variance^59^. In the PC analysis of this dataset, we kept the factor loadings over |0.35| of variables with significant contributions (*p* < 0.05) to reduce dataset dimensionality and to optimally separate animals of each group (Supplementary Table 1 and Supplementary Fig. 2 A). Next, in order to assess possible relationships between sets of variables within the same animals, PCA was performed on each set (n = 7) of variables, i.e., somatosensory and motor foot map features (n = 23 and 17 variables, respectively), neurotransmission (n = 4) and neurohistopathology (n = 6), as well as 3 more variables: locomotion at P30 (n = 24) and P65 (n = 24) and musculoskeletal histopathologies (n = 7) previously reported^23^. We thus obtained a score for each rat and for each set of variables along the PC that optimally differentiated the two groups of rats, depending on the set of variables (Supplementary Fig. 2B). The aim was to correlate the scores of each rat between the 7 sets of variables, i.e., the scores that optimally characterized each animal for a given set. We used these optimal scores to perform subsequent linear correlations (Pearson or Spearman correlations according to data normality) within the same animals by using R.

## Electronic supplementary material

Supplementary data
