# Supplementary material for: Early movement restriction leads to maladaptive plasticity in the sensorimotor cortex and to movement disorders
Source: Sci Rep. 2018 Nov 5;8:16328. doi: 10.1038/s41598-018-34312-y (PMC6218548; doi:10.1038/s41598-018-34312-y)
Supplement: Supplementary file 1 — Supplementary data [file 41598_2018_34312_MOESM1_ESM.docx]

**Supplementary data**

**Early movement restriction leads to maladaptive plasticity in the sensorimotor cortex and to movement disorders**

Authors: Maxime Delcour^a,†^, Michaël Russier^a,‡^, Francis Castets^b^, Nathalie Lorenzo^c^, Marie-Hélène Canu^d^, Florence Cayetanot^e^, Mary F Barbe^e^, Jacques-Olivier Coq^a,e,*^

**^a^** Neurosciences Intégratives et Adaptatives, UMR 7260, CNRS, Aix Marseille Université, 13331 Marseille, France

**^b^** Centre de Recherche en Neurobiologie et Neurophysiologie de Marseille UMR 7286, CNRS, Aix-Marseille Université, 13344 Marseille, France

**^c^** FR 3512 Fédération 3C, Aix Marseille Université – CNRS, 13331 Marseille, France

**^d^** Université de Lille, EA 7369 « Activité Physique, Muscle et Santé » - URePSSS - Unité de Recherche Pluridisciplinaire Sport Santé Société, 59000 Lille, France

**^e^** Institut de Neurosciences de la Timone, UMR 7289, CNRS, Aix Marseille Université, 13385 Marseille, France

**^e^** Department of Anatomy and Cell Biology, Lewis Katz School of Medicine, Temple University, Philadelphia, PA, 19140, USA.

**^*^** Correspondance to: Dr J-O Coq, Institut de Neurosciences de la Timone (INT) UMR 7289,Team P3M, 27 Bd Jean Moulin, Campus Santé Timone, 13385 Marseille Cedex 05, France; Tel : +33 491 32 40 25;Fax : +33 491 32 40 56; Email: [jacques-olivier.coq@univ-amu.fr](mailto:jacques-olivier.coq@univ-amu.fr)

Contents:

1. **Supplementary Methods**
2. **Supplementary Table 1**
3. **Supplementary Figure 1**
4. **Supplementary Figure 2**
5. **Supplementary Figure 3**
6. **Supplementary Figure 4**
7. **Supplementary methods**

All experiments and animal use have been carried out in accordance with the guidelines laid down by NIH (NIH Publication #80-23) and EU Directive (2010/63/EU). The research involving animals has been approved by the Direction Départementale des Services Vétérinaires – Préfecture des Bouches du Rhône, France (permit # C13-055-8JR). Every precaution was taken to minimize stress and the number of animals used in each series of experiments as our experimental activity was guided by the principle of the three R’s (Replacement, Reduction and Refinement).

**Electrophysiological mapping in cortical sensorimotor areas**

**Somatosensory maps**. Ten and nine electrophysiological maps of the hind limb skin representation were derived in the S1 cortex of control and SMR rats from a total amount of 1278 and 808 electrode recording sites, respectively, averaging 110 ± 28 (mean ± SD) cortical penetrations per map.

A craniotomy was made over the primary somatosensory cortex (S1) corresponding to the somatosensory hind limb representation. An enlarged image of the exposed brain surface was used to guide and record the location of electrode penetrations. Multiunit recordings were made using tungsten microelectrodes (1 MΩ at 1 KHz, WPI, Sarasota, FL, USA) in the upper layer IV (650-700 µm) of the S1 cortex. Electrodes were spaced 50-100 µm apart according to surface blood vessels. The multiunit signal was preamplified, filtered (bandpass, 0.5-5 KHz), displayed on an oscilloscope, and delivered to an audio monitor.

The receptive field (RF) corresponded to the cutaneous area whose barely visible indentation elicited clear bursts of S1 neuronal activity. Low-threshold cutaneous receptive fields were detected based on small skin indentations or gentle hair movements. High-threshold responses elicited by taps on the skin, stroking of hairs, and manipulations of muscles and joints were classified as non-cutaneous responses. Unresponsive cortical sites exhibited spontaneous discharges only. The ridges of glabrous skin of the toes and plantar surfaces were used as reliable landmarks to delineate RFs. The RF areas were transferred to the hind paw digital image then measured offline using MAP software. Deneba Canvas software (Victoria, BC, Canada) was used to elaborate maps of the hind limb representation by drawing boundaries encompassing cortical sites whose RFs were restricted to a common hind paw subdivision (e.g. toe, plantar pad). When RFs were located on distinct and separate skin subdivisions, borders were drawn midway between adjacent recording sites. When a single RF included different but adjoining skin subdivisions of the hind limb, a boundary line was drawn that crossed the cortical sites. Map borders were placed midway between responsive and unresponsive sites.

**Motor maps**. Cortical maps of the movement representation of the hind limb were derived in the primary motor cortex (M1) of 9 control and 8 SMR rats by intracortical microstimulation techniques. Maps were generated from a total of 1468 and 1334 stimulation sites in controls and SMR rats, respectively, corresponding to 164 ± 34 cortical sites per maps on average.

After craniotomy in the M1 hind limb area, an enlarged image of the exposed brain surface was used to guide and to record the location of electrode penetrations. Microelectrodes (100 KΩ, FHC, Bowdoin, ME, USA) were advanced into layer V at a depth of about 1700 µm. Electrodes were spaced 100-200 µm apart at the cortical surface according to vasculature. Microstimulations consisted of 40-ms trains of 200-µs biphasic cathodal pulses delivered at 333 Hz by an electrically isolated constant-current stimulator (A-M Systems, Sequim, WA, USA). Pulse trains were delivered at about 1 Hz (Strata et al., 2004). At each electrode penetration, stimulation was initiated at the lowest intensity (5 µA) and was increased gradually until lower limb movement was evoked. Movements of the toes, ankle, knee, hip, and other joints were detected visually. If no movement was elicited at 60 µA, the site was defined as unresponsive. The motor hind limb area was delimited by either no responses or by whisker, jaw or neck movements in the medial, caudal and lateral borders and by forelimb movements in the rostral border. Maps were reconstructed by drawing boundaries encompassing cortical sites whose stimulation elicited movements of the same joint (hip, knee, ankle, toe, and multi-joint complex movements).

**Excitatory and inhibitory neurotransmission**

**In vivo microdialysis**. Carnegie Medecin microdialysis probes (CMA/11, Phymep, France) were implanted within the hindpaw representation of the right S1-M1 area. Each rat was dialysated two hours without collecting, in order to reach a basal neurotransmitter level after the introduction of the probe. Dialysis probe was perfused at 2 µL.min^-1^ using a microinjection pump (CMA/100, Phymep), with an artificial CSF containing (in mM): NaCl = 147; KCl = 2.7; CaCl2 = 1.2; MgCl2 = 0.85 (CMA, Phymep). Dialysates were collected every 5 min (10 µL by sample) on a Univentor refrigerate microfraction collector (Phymep) during 30 min and stocked at –80 °C before analysis.

The extracellular concentrations of glutamate (GLU) and GABA in the dialysates were determined by gradient High Performance Liquid Chromatography (Dionex, France) coupled with laser detection (Picometrics, France). Pre-column derivation consisted in a NDA (2,3Naphthalenedicaboxaldehyde) derivation. Thirty min before analysis, a solution F freshly synthesised (20 µL) containing 1.6 mL of Solution E (in 100 mL of distilled water) = Sodium tetraborate + Boric Acid; 400 µL of Solution D (in 10 mL of distilled water) = 28 mg Potassium Cyanide and 333 µL of Solution C (in 10 mL of distilled water and acetonitrile in the same proportion) = 9.2 mg of NDA was added to each sample (10 µL). During these 30 min, the samples were maintained at room temperature, in the dark. Following these procedures, 8 µL of the 30 µL derived sample were injected into a C18 column (300 µm x 150 mm, i.d.; particle size 3 µm; Dionex) eluted with buffer 1 which contained citrate sodium 0.05 adjusted to pH 3.5 and buffer 2 containing 100% Acetonitrile. The concentration gradient was established for the best separation of GLU and GABA. The retention time was 13.5 min and 19.5 min for GLU and GABA, respectively. The column was perfused at 0.7 mL.min^-1^ and maintained at 25°C. Detection was performed by a laser (LIF, Picometrics) and the signal was integrated using a Dionex Integration Pack.

**Western blotting and quantification.** Western blot quantification was performed on scanned autoradiographies with Image J software (Abramoff et al., 2004). Integrative intensities minus background were plotted for each sample after normalization to 1 for the highest value for one western blot. Original pictures of the membranes after transfer and revelation with specific antibodies are shown in Supplementary Fig. 4.

Mouse monoclonal anti-tubulin (E7) antibody, from the Developmental Studies Hybridoma Bank (Iowa city, IA, USA), was used at 0.2 µg.mL^-1^. Mouse monoclonal anti-vGLUT1 antibody (clone N28/9, NeuroMab, UC Davis, CA) was used at 1 µg.mL^-1^. Rabbit polyclonal anti-vGAT antibody (#AB5062P Millipore, Temecula, CA) was used at 1µg.mL^-1^.

**Brain immunohistology**

After terminal anaesthesia (thiopentobarbital, 150 mg.Kg^-1^), each animal underwent transcardial perfused with 4% paraformaldehyde in phosphate buffer (0.10 M, pH 7.4). Whole brains were collected, postfixed for 24 hours in the same fixative, and then equilibrated in 10%, then 20%, and then 30% sucrose for 24 hours each before freezing and storing at -80^o^C until use. Brain sections to be immunostained for glial fibrillary acidic protein (GFAP), amyloid-ß precursor protein (APP), DAPI (a general cell nucleus marker), NeuN (a neuronal nuclear marker) and GAD (GABAergic interneurons expressing GAD67) consisted of 15 µm coronal serial cryosections. that had been mounted onto Fisher Plus slides (Fisher Scientific, Pittsburgh, PA, USA), dried overnight on the slides at room temperature, washed in 0.1 M phosphate-buffer saline with 0.02% sodium azide (PBS, pH 7.6/7.4) for 10 min, and then in 10% goat serum for 20 min at room temperature (with 0.01% triton for APP. The goat serum step was preceded by 0.3% H_2_O_2_ in methanol for 20 min), and then alternating sections stained for the various primary antibodies. Primary antibodies were incubated overnight at the appropriate dilution in PBS at room temperature (see Table below), and visualized using the appropriate secondary antibody conjugated to Cy3 and Cy2 (red and green fluorescence, respectively; 1:100 in PBS, using a 2 h incubation at room temperature; Jackson ImmunoResearch, Westgrove PA, USA) (see (Delcour et al., 2012a, 2012b, 2011) for details).

Table of primary antibodies used for brain immunohistology:

| Marker | Manufacturer | Dilution |
| --- | --- | --- |
| APP | Sigma-Aldrich (St Louis, MO, USA) | 1/500 |
| Caspase 3 | Millipore (Temecula, CA, USA) | 1/250 |
| GAD67 | Chemicon (Temecula, CA, USA) | 1/2000 |
| GFAP | Chemicon (Temecula, CA, USA) | 1/1000 |
| NeuN | Chemicon (Temecula, CA, USA) | 1/2000 |

GFAP immunoreactive cell counting (cell density) was performed using epi-fluorescence with a 40x objective in every 10^th^ serial section in the area of interest within a 0.065 mm^2^ rectangular region in 3 sections per animal, and with 6 or more animals per group. To avoid bias, only GFAP+ cells in which the nucleus (unlabeled or DAPI labelled) was visible was measured. APP was visualized using an anti-rabbit secondary antibody with a horseradish peroxidase tag (1:100 for 2 h in PBS, Jackson Immuno-Research) followed by reacting the peroxidase with H_2_O_2_ (0.003%) in the presence of 0.02% 3,3'-diaminobenzidine tetrahydrochloride (DAB; brown staining) in 0.05 M TRIS buffer. Quantification was performed using a E800 microscope (Nikon, Melville, NY, USA) equipped with a motorized stage and the Bioquant System software-controlled computer system (Bioquant Image Analysis Corp, Nashville, TN, USA) linked to a Retiga EXI cooled camera (QImaging, Surrey, BC). The mean area fraction of immunoreactive product in the field (using a 40 X objective and the irregular region of interest tool of the Bioquant software so as to remain within the appropriate cortical or white matter region) was determined by dividing the videocount area of pixels with staining at or above a user-defined background threshold by the videocount area of all pixels in the field. The same background threshold was used for all APP measurements. The percent area fraction with APP immunoreactive product was quantified in every 10^th^ serial section, in the area of interest, in 3 sections per animal, and with 6 or more animals per group. All GFAP and APP counts were performed on coded preparations by one blinded observer.

Counting of NeuN and GAD-67 cells was performed using a DMR microscope (Zeiss, Oberkochen, Germany) equipped with a motorized stage and Stereo Investigator software-controlled computer system (Microbright-Field Europe, Magdeburg, Germany). Cell counting was performed at x40 magnification with the optical fractionator method in semi serial sections. The areas of the S1 and M1 cortices were circumscribed at x1.25 magnification on live microscopic images displayed on a monitor. The software program combines optical dissector counting with fractionator sampling. Uniform grid areas of 200 x 200 μm² and 100 x 100 μm² were placed over M1 and S1 on sections labelled with NeuN and GAD-67. The software program automatically and randomly moves across sampling areas (optical dissector) that include appropriate “acceptance” and “forbidden” lines. The size of these sampling areas was 50 x 50 μm². We also evaluated section thickness after staining the sections with an antibody directed against the mature neuronal marker NeuN. Mean section thickness was 20 ± 1.5 μm. Only cells that fell within the sampling area or touched the “acceptance” line were counted. Similarly, a cell was counted only if the bottom of the cell was visible within the volume of the dissector (height of section thickness excluding 2 μm of thick guard zone). In both groups, the number of samples from the left and right hemispheres was roughly equal.

1. **Supplementary Table 1.**

Description of all the variables (n = 105) used in principal components (PC) analysis and color-coded representation of significant factor loadings. *Left column*. Description of the variables in each set of variables (n = 7), e.g., locomotion at P30 or P65, musculoskeletal histopathology (described previously in (Delcour et al., 2018), somatosensory and motor map features, excitation/inhibition neurotransmission and neurohistopathology. *Middle column*. Color-coded illustration of the factor loadings associated with PC1, i.e., the correlations between each variable and the PC1 or axis, corresponding to a coordinate for each variable along this axis. Note that we only kept the variables whose factor loading was significantly correlated with PC1, i.e., with a color code in red or blue, along with color intensity according to correlation magnitude. *Right column*. Description of the variables that were significantly correlated to PC2. Note also that the factor loadings of most of the motor map features were significantly correlated along the PC2 while a very few ones to PC1. The biplot representation of the significant variables with factor loadings > |0.35| is depicted in Supporting Information Figure S1B.

1. **Supplementary Figure 1.**

Images of APP, caspase 3 and GFAP immunoreactivity of the corpus callosum and cingulate cortex white matter in control rats and in rats exposed to postnatal sensorimotor restriction (SMR). Note the absence of differences in immunostaining between the two groups of rats, indicative of an absence of brain pathology after SMR. Scale bars: 50 µm


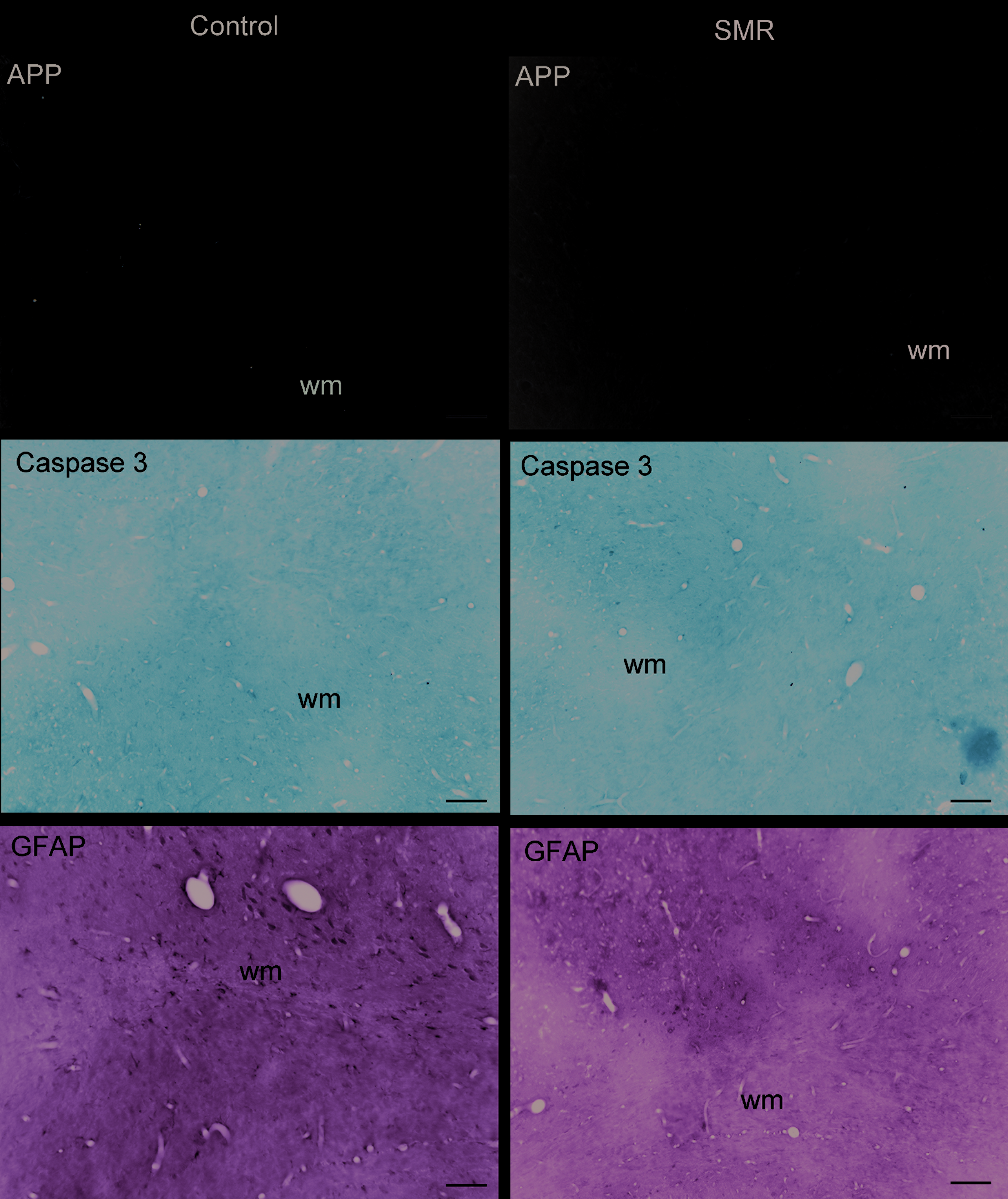


1. **Supplementary Figure 2.**

Illustration of the two methods used to compute the individual rat’s scores for the variables based on principal components (PC) analysis (PCA). **A.** The first method of PCA was aimed at pointing out the variables (n=54 variables, see Table S1, left column) among all (n=105) that contributed the most to optimally differentiate the two groups of rats (see text). In the biplot of control (black dots) and SMR (red dots) rats after PCA on the whole dataset, note the significant separation (*p* < 0.0001) of the two groups along PC1, emphasized by the barycentres of each group of rat. Note that 24.4% of all the dataset was summarized along the PC1 or x-axis, while the two groups of rats were not differentiated along PC2 (9.9% of variance summarized), so that PC2 was not used (see Supplementary Table 1). **B.** The second method of PCA is used to compute the relationships between the 7 sets of variables (all variables and the corresponding sets are described in Supplementary Table 1) for each animal. PCA was performed on the variables of each set to obtain a score according to the optimal PC or axis that best differentiated the two groups of rats, depending on the variables. This method has the advantage to reduce the dimensionality to obtain a single score for each animal in every set of variables. The score for each animal was linearly correlated with the same animal’s scores from other sets of variables. *Left panel.* Color-coded representation of the factor loading for each variable in the first two PCs for the 7 sets of variables used in the previous (Delcour et al., 2018) and present study. *Right panel*. Biplot representations of each rat (denoted by filled dots) according to the factor loadings of variables along the two PCs or axes in each set of variables, illustrated in the left column. The scores or coordinates along each PC summarized the percentage of variance of the corresponding set of variables, maximizing the variance and minimizing the loss of information. Note the significant differentiation of the two groups of rats along the PC1 or x-axis for locomotion at P30, at P65, musculoskeletal histopathology and the S1 map features, emphasized by the barycentres of each group of rat (denoted by filled or empty squares). The optimal and significant separation of the two groups for the M1 map features was along the PC2 or y-axis. The PCA performed on the 7^th^ set of variables on neurohistopathology are not depicted because no variable correlated with any axis.

**
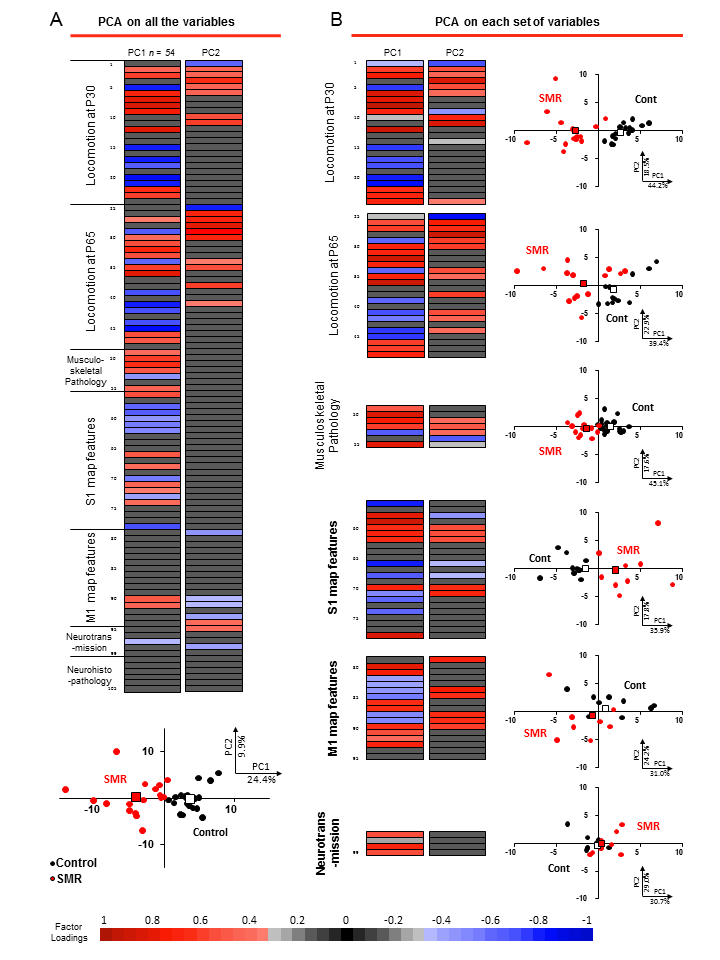
**

1. **Supplemental Figure 3.**

Overlap between somatosensory and motor hind limb maps in control and SMR rats. The boundaries of somatosensory and motor maps in 4 control rats *(upper panel)* and of both maps in 4 SMR rats *(lower panel*) are depicted in reference to the position of bregma. Note the overall overlap between somatosensory and motor maps whether of the group of rats.


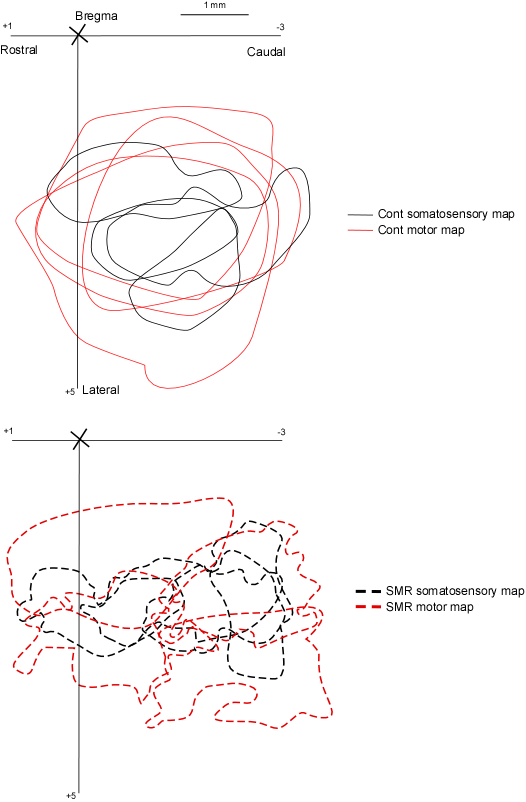


1. **Supplementary Figure 4.**

Original pictures used to construct the figure 3. **A.** Ponceau red staining of nitrocellulose membrane used for vGlut and tubulin revelation. **B.** Original scan of the film obtained with vGlut1 antibody. **C.** Original scan of the film obtained with tubulin antibody. **D.** Ponceau red staining of nitrocellulose membrane used for vGAT revelation. **E.** Original scan of the film obtained with VGAT antibody.

**References**

Abramoff, M.D., Magalhães, P.J., Ram, S.J., 2004. Image processing with ImageJ. Biophotonics Int. 11, 36–42.

Delcour, M., Massicotte, V., Russier, M., Bras, H., Peyronnet, J., Canu, M.-H., Cayetanot, F., Barbe, M.F., Coq J.O., 2018. Early movement restriction leads to enduring disorders in muscle and locomotion. Brain Pathol. https://doi.org/10.1111/bpa.12594

Delcour, M., Olivier, P., Chambon, C., Pansiot, J., Russier, M., Liberge, M., Xin, D., Gestreau, C., Alescio-Lautier, B., Gressens, P., Verney, C., Barbe, M.F., Baud, O., Coq, J.O., 2012a. Neuroanatomical, Sensorimotor and Cognitive Deficits in Adult Rats with White Matter Injury following Prenatal Ischemia. Brain Pathol. 22, 1–16.

Delcour, M., Russier, M., Amin, M., Baud, O., Paban, V., Barbe, M.F., Coq, J.O., 2012b. Impact of prenatal ischemia on behavior, cognitive abilities and neuroanatomy in adult rats with white matter damage. Behav. Brain Res. 232, 233–244.

Delcour, M., Russier, M., Xin, D., Massicotte, V.S., Barbe, M.F., Coq, J.O., 2011. Mild musculoskeletal and locomotor alterations in adult rats with white matter injury following prenatal ischemia. Int. J. Dev. Neurosci. 29, 593–607.

Strata, F., Coq, J.O., Byl, N., Merzenich, M.M., 2004. Effects of sensorimotor restriction and anoxia on gait and motor cortex organization: implications for a rodent model of cerebral palsy. Neuroscience 129, 141–156.
